# Structures of NHBA elucidate a broadly conserved epitope identified by a vaccine induced antibody

**DOI:** 10.1371/journal.pone.0201922

**Published:** 2018-08-22

**Authors:** Martina Maritan, Daniele Veggi, Roberta Cozzi, Lucia Dello Iacono, Erika Bartolini, Paola Lo Surdo, Giulietta Maruggi, Glen Spraggon, Matthew J. Bottomley, Enrico Malito

**Affiliations:** 1 GSK, Siena, Italy; 2 GSK, Rockville, MD, United States of America; 3 Genomics Institute of the Novartis Research Foundation, San Diego, CA, United States of America; Universidad Nacional de la Plata, ARGENTINA

## Abstract

Neisserial heparin binding antigen (NHBA) is one of three main recombinant protein antigens in 4CMenB, a vaccine for the prevention of invasive meningococcal disease caused by *Neisseria meningitidis* serogroup B. NHBA is a surface-exposed lipoprotein composed of a predicted disordered N-terminal region, an arginine-rich region that binds heparin, and a C-terminal domain that folds as an anti-parallel β-barrel and that upon release after cleavage by human proteases alters endothelial permeability. NHBA induces bactericidal antibodies in humans, and NHBA-specific antibodies elicited by the 4CMenB vaccine contribute to serum bactericidal activity, the correlate of protection. To better understand the structural bases of the human antibody response to 4CMenB vaccination and to inform antigen design, we used X-ray crystallography to elucidate the structures of two C-terminal fragments of NHBA, either alone or in complex with the Fab derived from the vaccine-elicited human monoclonal antibody 5H2, and the structure of the unbound Fab 5H2. The structures reveal details on the interaction between an N-terminal β-hairpin fragment and the β-barrel, and explain how NHBA is capable of generating cross-reactive antibodies through an extensive conserved conformational epitope that covers the entire C-terminal face of the β-barrel. By providing new structural information on a vaccine antigen and on the human immune response to vaccination, these results deepen our *molecular* understanding of 4CMenB, and might also aid future vaccine design projects.

## Introduction

*Neisseria meningitidis* is a gram-negative bacterium and a leading cause of bacterial meningitis and sepsis [1], with highest incidence in infants and adolescents [2]. Glycoconjugate vaccines that protect against disease caused by meningococci serogroups A, C, W and Y have been available since the 1990s [3]. More recently, two vaccines have been developed to protect against serogroup B meningococcus (MenB), which is responsible for the majority of invasive meningococcal disease in developed countries. These vaccines are known as 4CMenB (*Bexsero*, GSK), which is made of three main recombinant protein antigens, NHBA, NadA, and fHbp, plus outer membrane vesicles (OMV) [4], and as rLP2086 (Trumenba, Pfizer), which is composed of two fHbp variants. *Bexsero* has been licensed in Europe, Australia, Canada, and Latin America for use in all age groups from 2 months [5]. Bexsero is a trade mark of the GSK group of companies.

This study focuses on the 4CMenB antigen NHBA (Neisserial Heparin Binding Antigen), also known as genome-derived neisserial antigen 2132 (GNA2132). The *nhba* gene is ubiquitous in meningococcal strains of all serogroups, and is also found in *N*. *gonorrhoeae* and other commensal Neisserial species (*Neisseria lactamica*, *Neisseria polysaccharea*, and *Neisseria flavescens*) [6]. The gene product is a surface-exposed protein of around 420–490 amino acids [7]. Over 400 different peptide variants of NHBA have been reported, which are mainly cross-protective [6]. 4CMenB contains NHBA peptide 2 (p2) as the N-terminal part of a fusion protein with an additional antigen, NMB1030, a ubiquinone-8 binding protein also known as NUbp [8, 9]. For this study, we focused on fragments and constructs of NHBA peptide 20.

**Fig 1:**
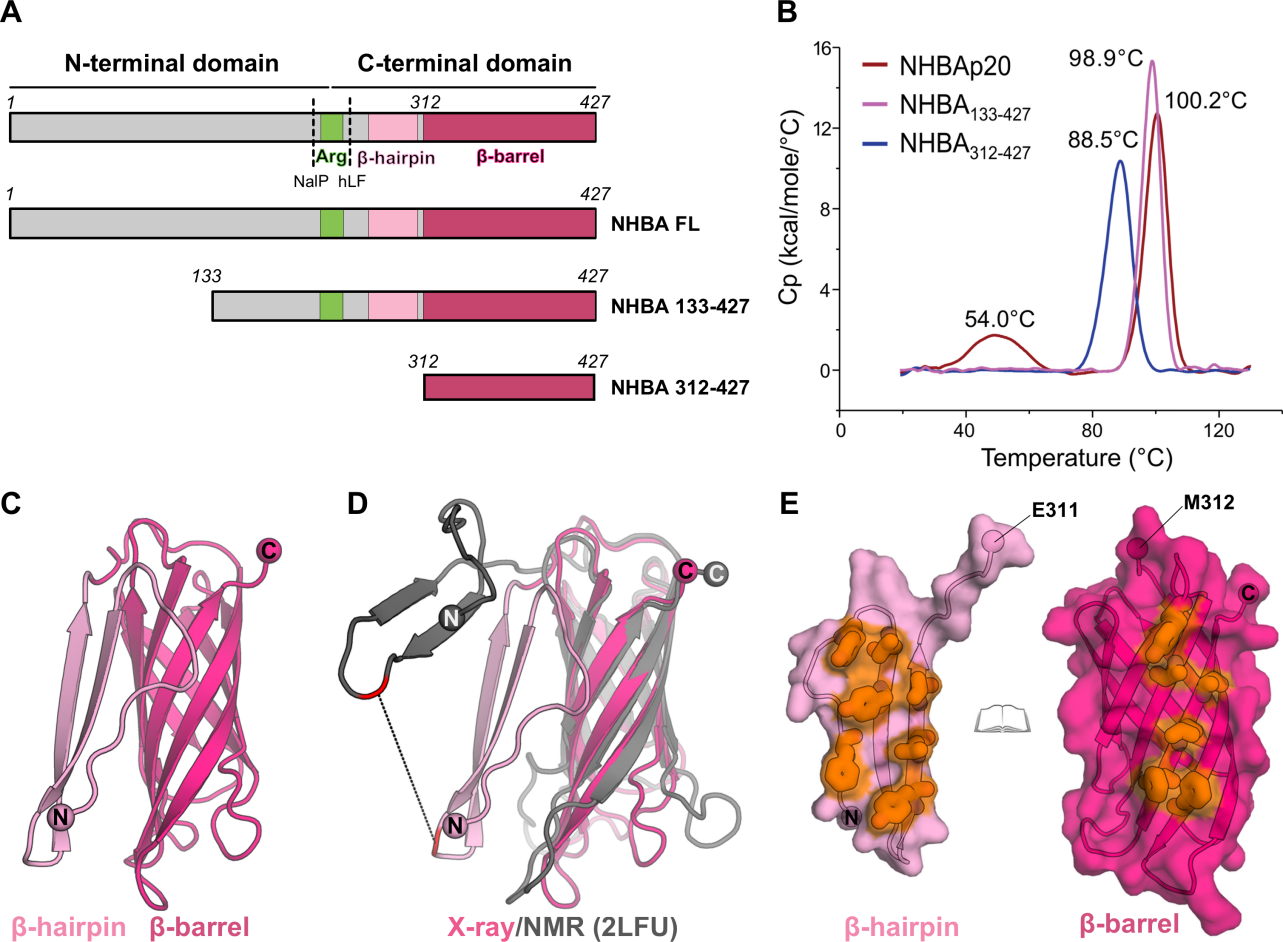
Domain organization, thermo-stability, and structure of NHBA. **A**) The constructs of NHBA studied here are shown and compared to the full-length protein. Domains are depicted as schematic boxes and labeled. NalP and Human Lactoferrin (hLF) cleavage sites are shown with dashed lines and labeled. **B)** DSC profiles of full-length NHBAp20 (red), NHBA_133-427_ (pink), and NHBA_312-427_ (blue). *T*_m_ values of each peak are labelled. **C)** The crystal structure of NHBA_133-427_ is shown as cartoon. **D**) Superposition of one monomer of the NHBA_133-427_ crystal structure (light and dark pink cartoon as in A) onto the one of the ten model from the NHBA NMR structure (pdb 2LFU, dark grey). A black dashed line connects common residues P295. **E**) Open-book view of the interface between the β-hairpin (light pink, left) and the β-barrel (dark pink, right), with hydrophobic residues that make the interface colored in orange, several Phe and Tyr side chains are shown as thick sticks.

The NHBA protein has an N-terminal region of approximately 250 residues predicted to be intrinsically disordered, as previously reported [10] and calculated with Phyre2 [11], SABLE [12], PSIPRED [13] and JPred [14], and a highly conserved C-terminal domain (approx. 180 residues). Solution NMR studies of NHBA residues 245–427 (strain 2996, NHBA peptide p20) revealed a single 8-stranded anti-parallel β-barrel with a variably-positioned N-terminal extension made of an hairpin of two β-strands [15]. In a small central region between the N- and C-terminal regions, NHBA binds heparin and heparan sulfate through an Arg-rich motif [7], and this ability has been shown to increase bacterial serum resistance and epithelial cell binding [16]. Meningococcal NalP protease and human lactoferrin (hLf) are capable of cleaving NHBA upstream and downstream of the 10-residue Arg-rich region, respectively, [7] (**Fig 1A**). Upon cleavage by NalP, a C-terminal NHBA fragment is released and can alter endothelial permeability, likely contributing to the vascular leakage that is typically associated with meningococcal sepsis [17].

A study of antibody responses induced upon 4CMenB vaccination has been recently published [18], where a panel of human monoclonal antibodies (humAbs) from adult vaccinees was isolated, and antigen-specific monoclonal antibodies (mAbs) were identified and produced as recombinant fragment antigen-binding domains (Fabs). Seven unique rearrangements were identified among the twenty-five humAbs specific for NHBA isolated from two immunized adult subjects, of which four recognizing the C-terminal β-barrel domain, two the N-terminal domain and one not showing appreciable binding to NHBA. Interestingly, humAbs binding to the C-terminal domain displayed higher binding affinities and were also more cross-reactive than those targeting the N-terminal region. Also, anti-NHBA antibodies contribute to serum bactericidal antibody (SBA) elicited by 4CMenB [19].

In addition to the original aim of increasing the structural knowledge of NHBA, the observations above prompted us to investigate the molecular bases for the recognition of conserved NHBA epitopes by cross-reactive human high-affinity antibodies. Here, we first solved the crystal structure of an NHBA fragment that reveals a previously unseen conformation of a 2-stranded ß-hairpin packing against the C-terminal domain. Then, we solved the crystal structures of the NHBA fragment 312–427 in complex with the Fab fragment of the humAb 5H2, and of the apo (unbound) huFab 5H2, revealing the presence of a large cross-reactive conformational epitope on NHBA and elucidating antibody plasticity that is required for binding to NHBA. Implications regarding the human immune response to the 4CMenB vaccine are discussed.

## Materials & methods

### NHBA_133-427_ and NHBA_312-427_ cloning, expression and purification

Constructs NHBA_133-427_ and NHBA_312-427_, which include residues A133-D427 and M312-D427, respectively, were derived from NHBA peptide 20 (*N*. *meningitidis* strain 2996 [20] Uniprot Q9JPP1). The NHBA gene fragment encoding amino acids 133–427 was PCR amplified and then inserted, using the polymerase incomplete primer extension (PIPE) cloning method [21], into the pET-21b(+) expression vector (Novagen) to enable isopropyl β-D-thiogalactopyranoside (IPTG)-inducible expression. The construct was cloned after an MA (Met, Ala) sequence, added for cloning purposes, and was followed by a C-terminal hexahistidine (His6) tag (residues LEHHHHHH) for purification purposes. The construct and NHBA gene fragment were confirmed in-house by DNA sequencing prior to protein production. The NHBA_312-427_ construct was cloned in a pET-21b+ vector (Novagen) and using a C-terminal His6 tag. Site-directed mutagenesis on NHBA_312-427_ DNA sequences was performed to produce seven mutants using the PIPE method. The resulting constructs were: R339A, R339G, K367A, R339A-K367A, R339G-K367A, T365A-D360A and E322A-E425A. The mutagenesis reaction products were transformed into chemically competent *E*. *coli* Mach1^TM^-T1R (Thermo Scientific).

The expression of the NHBA_133-427_ and NHBA_312-427_ proteins was performed using *Escherichia coli* strain BL21 (DE3) cells (Novagen). In both cases, cells were grown using using BioSilta medium (Enpresso B Animal-free growth systems) supplemented with 100 μg/mL ampicillin, and following the manufacturer’s instructions. Cell cultures were aerated in 250 mL shake flasks at 37°C for 20-hours (NHBA_133-427_), or at 30°C for 12 h (NHBA_312-427_), and the production of proteins was induced by the addition of 0.1 mM IPTG. After an additional 24 h culture, cells were harvested by centrifugation.

Purifications of both NHBA_133-427_ and NHBA_312-427_ were performed using a sonication protocol for the lysis followed by Co^2+^-affinity chromatography (HiTrap TALON crude, GE Healthcare) *via* C-terminal His6 tags and a size exclusion chromatography (SEC) step (Superdex 75 16/60, GE Healthcare) in 20 mM Tris, 150 mM NaCl pH 8.0 buffer. The final sample purity and concentration were assessed by SDS-PAGE after Coomassie staining and by absorbance measurements at 280 nm. The quality of the final NHBA_133-427_ samples was also checked by size-exclusion high-performance liquid chromatography coupled with multi-angle laser light scattering (SE-HPLC/MALS). Samples were eluted isocratically in phosphate-buffered saline (PBS) buffer at pH 7.5. Data analyses were carried out using Astra V software (Wyatt) to determine the weight-average molecular mass (MW) in Daltons and the polydispersity index (MW/Mn) for each oligomer present in solution.

### Recombinant Fab 5H2 expression and purification

Monoclonal antibody 5H2 was raised by human immunization with the 4CMenB vaccine formulation containing the fusion antigen NHBA-NMB1030, via a Phase I clinical trial conducted in Krakow, Poland, approved by the Bioethics Committee of the District Medical Doctors’ Chamber in Krakow and conducted in accordance with the Declaration of Helsinki. This study was approved by the sponsor internal ethics review board and local ethics committees and conducted according to good clinical practice in accordance with the declaration of Helsinki. The use of samples was performed upon written informed consent obtained from participants before the study-specific procedures. We wish to thank the clinical study participants. Sequences of the variable regions of 5H2 were identified as previously described [18]. Gene fragments corresponding to the heavy and light chains of the variable regions were synthesized by Geneart (Life Technologies) with Eco31I site flanking gene extremities. The 5H2 Fab was produced by transient transfection using Expi293 (Life Technologies) cells in suspension, according to the manufacturer’s protocol. Equal amounts (15 μg each/30 mL of transfection volume) of vector DNA encoding FabH and FabL chain were used to transfect Expi293 cells. The cells were incubated at 37°C with a humidified atmosphere of 8% CO2 in air on an orbital shaker rotating at 125 rpm. Cell culture supernatant was harvested after 36 and 72 h post transfection, clarified by centrifugation for 30 min at 4000 rpm and concentrated. The Fab was purified from the supernatant using a Strep Trap HP pre-packed column with StrepTactin Sepharose High Performance medium (GE Healthcare), followed by gel filtration chromatography on a Superdex 200 16/60 column (GE Healthcare). After purification, a cleavable C-terminal Strep tag II on the heavy chain was removed with TEV (tobacco etch virus) protease. Purity of the Fab was assessed by sodium dodecyl sulfate polyacrylamide gel electrophoresis (SDS-PAGE) after Coomassie staining and its amount was quantified by absorbance at 280 nm.

### Protein crystallization and X-ray data collection

Samples of NHBA_133-427_ were concentrated to 24 mg/mL and immediately used for crystallization experiments. The complex Fab5H2:NHBA_312-427_ was freshly prepared by co-incubation at 4°C overnight, followed by preparative SEC in 20 mM Tris-HCl, 150 mM NaCl, pH 8.0 using Superdex 200 16/60 (GE Healthcare), and used at a concentration of 5 mg/mL for crystallization experiments. Finally, samples of unbound Fab 5H2 were used at a concentration of 9 mg/mL. All samples were concentrated using centrifugal concentration devices with 10 kDa molecular weight cut-off membrane (Amicon Ultra-15 Centrifugal Filter Unit, Millipore).

Crystallization trials were performed using 96-well low-profile Intelli-Plates (Art Robbins) and a nanodroplet sitting-drop vapour-diffusion format by mixing equal volumes (200 nL) of protein samples and crystallization buffers with a Crystal Gryphon liquid dispenser (Art Robbins Instruments). Experiments were performed at 20°C and crystallization trays were imaged with the automatic imaging system RockImager-182 (Formulatrix). One single crystal of NHBA_133-427_ grew after 6 months from a drop containing 20% PEG 3,350, 0.2 M Sodium thiocyanate. This crystal diffracted X-rays at a resolution of 1.8 Å, and the diffraction data were collected on beamline ID23-1 at the European Synchrotron Radiation Facility (ESRF, Grenoble, France), revealing space group *P*4_1_, with a calculated Matthews coefficient of 2.49 Å^3^/Da and a solvent content of 50%. Crystals of unbound Fab 5H2 were obtained in 0.2 M (NH_4_)_2_SO_4_, 0.1 M Na-cacodylate pH 6.5 and 30% w/v PEG 8K and diffracted X-rays to a resolution of 1.88 Å. The data were collected on beamline ID29 at ESRF, revealing the monoclinic space group *P*2_1_, with a calculated Matthews coefficient of 2.29 Å^3^/Da, which correspond to a solvent content of 46.3% and three Fab molecules in the asymmetric unit. Crystals of the complex Fab5H2:NHBA_312-427_ grew in 0.1 M TRIS pH 8.0 and 1.6 M Li_2_SO_4_, diffracted to a resolution of 2.8 Å, and the diffraction data were collected on beamline I02 at the Diamond Light Source (Oxford, UK). These complex crystals belonged to space group *P*6_1_22, with two copies of the complex in the asymmetric unit, for a calculated Matthews coefficient of 2.91 Å^3^/Da and a solvent content of 62.6%.

Prior to data collection, all crystals were cryo-protected in mother liquor supplemented with 15% glycerol and flash frozen in liquid nitrogen. All diffraction data were processed with iMosflm [22], XDS [23] AIMLESS [24] and programs of the CCP4 software suite [25].

### Structures determination and refinement

The structure of NHBA_133-427_ was solved by molecular replacement (MR) in Phaser [26] using as input model template the coordinates of one single model randomly selected from the NMR structure ensemble previously deposited in the Protein Data Bank (PDB 2LFU) [15]. Excellent electron density could be observed allowing manual model building with Coot [27] for residues N275-Q426 of chain A and I277-D427 of chain B, for a total number of 301 residues. Refinement was performed with BUSTER [28] and phenix (Adams, 2010 #325), to final *R*_work_/*R*_free_ values of 19.2/23.8% (**Table 1**).

**Table 1:** Data collection and refinement statistics.

	NHBA_133-427_ (PDB 6CUJ)	Fab 5H2 (PDB 5NYX)	Fab 5H2:NHBA_312-427_ (PDB 5O1R)
**Wavelength**	0.96862	0.97721	0.97950
**Resolution range**	46.1–1.8 (1.86–1.80)	47.0–1.88 (1.94–1.88)	78.7–2.8 (2.96–2.86)
**Space group**	*P* 4_1_	*P* 1 2_1_ 1	*P* 6_1_ 2 2
**Unit cell, dimensions** **angles**	77.0, 77.0, 57.5 90, 90, 90	82.0, 105.7, 86.2 90, 111.9, 90	119.4, 119.4, 364.2 90, 90, 120
**Total reflections**	144731 (23727)	325510 (32667)	710332 (50746)
**Unique reflections**	31127 (5025)	109353 (10952)	36576 (3562)
**Multiplicity**	4.6 (4.7)	3.0 (3.0)	19.4 (19.3)
**Completeness (%)**	99.9 (99.9)	99 (99.2)	100 (100)
**Mean I/sigma(I)**	13.4 (2.3)	6.6 (1.3)	18.4 (1.0)
**Wilson B-factor (Å**^**2**^**)**	34.3	29.8	80.0
**R-merge**	6.2 (47.2)	8.2 (83.1)	18.9 (376.5)
**R-meas**	6.9 (53.1)	10.0 (100.8)	19.4 (386.6)
**CC 1/2**	99.9 (84.7)	99.4 (47.4)	99.9 (55.7)
**Reflections used in refinement**	31105 (2664)	109345 (10952)	36559 (3559)
**Reflections used for R-free**	1555 (141)	5540 (534)	1775 (182)
**R-work**	19.1 (26.2)	18.6 (28.7)	19.2 (33.93)
**R-free**	23.8 (29.4)	22.6 (34.2)	23.84 (37.50)
**Number of non-H atoms**	2367	10613	8326
**Macromolecules**	2246	9805	8260
**Ligands**	-	82	20
**Solvent**	121	726	46
**Protein residues**	301	1291	1100
**Rms(bonds)**	0.007	0.007	0.014
**Rms(angles)**	0.897	0.94	1.93
**Ramachandran favored (%)**	96.2	98	96
**Ramachandran allowed (%)**	3.0	2.1	3.2
**Ramachandran outliers (%)**	0.6	0	0.55
**Rotamer outliers (%)**	0	1.3	11
**Clashscore**	5.05	7.65	5.45
**Average B-factor**	31.8	34.6	84.5
**Macromolecules**	31.5	34.0	84.6
**Ligands**	-	50.3	91.5
**Solvent**	40.4	40.6	67.9

*Values in parentheses are for the outer shell*.

R_*work*_ *= Σ||F(obs)| − |F(calc)||/ Σ|F(obs)|*

R_*free*_ *=* R_*work*_ *but calculated for 5% of the total reflections*, *chosen at random*, *and omitted from refinement*.

The structure of unbound Fab 5H2 was solved by molecular replacement using the software package MoRDa [29], using the coordinates of the anti-HIV-1 A32 Fab and of the anti-integrin LFA-1 FabAL-57 (PDBs 3TNM and 3HI6) as input template search models. Refinement and rebuilding were performed with BUSTER [28] and Coot [27], using non-crystallographic symmetry (NCS) restraints. Excellent electron density was observed for residues Q1-K224 and E1-G211 of the H and the L chains, for residues V2-K224 and E1-R210 of M and N chains and for Q1-K224 of the O chain, respectively. Chain P made an exception showing good electron density for the whole chain (residues E1-G211), with the exception of residues T48-R61, that were not traceable in the electron density map. Residues Y49-S60 of the light chain P were not modeled due to lack of density. The final model was refined to *R*_work_/*R*_free_ values of 18.6% and 22.6%, respectively (**Table 1**).

The structure of the Fab5H2-NHBA_312-427_ complex was solved by molecular replacement with Phaser [26], using the refined coordinates of the previously solved unbound Fab 5H2 and trimmed NHBA_133-427_ structures as input search model templates. Refinement and rebuilding were performed with Phenix [30] and Coot [31], to final *R*_work_ and *R*_free_ values of 19.1% and 23.8% (**Table 1**). Excellent electron density could be observed for almost the entire complex (residues M312-D427 for NHBA_312-427_, residues Q1-C226 and E1-E212 for the Fab 5H2 H and L chain, respectively). The only regions that could not be modeled due to lack of electron density were those of residues A410-E411 and of the C-terminal His-tag of both NHBA_312-427_ molecules present in the asymmetric unit.

The atomic coordinates and the structure factors of the three X-ray structures have been deposited in the Protein Data Bank with the following accession numbers: 6CUJ (NHBA_133-427_), 5O1R (Fab 5H2:NHBA_312-427_), 5NYX (Fab5H2). The quality of each structure was assessed using MolProbity [30]. Fab elbow angles were calculated with phenix.fab_elbow_angle [30]. Protein-protein interfaces were analyzed and calculated using the Protein Interfaces, Surfaces and Assemblies service (PISA) available at the European Bioinformatics Institute (http://www.ebi.ac.uk/msd-srv/prot_int/pistart.html). Structural comparisons were performed using the Secondary Structure Matching (SSM) algorithm within Coot [31]. Figures were generated using PyMOL (http://www.pymol.org/).

### Differential scanning calorimetry

The thermal stability of NHBA proteins was assessed by differential scanning calorimetry (DSC) using a MicroCal VP-Capillary DSC instrument (GE Healthcare), with a temperature scan range from 10°C to 130°C, a thermal ramp rate of 150°C per hour and a 5 second filter period. All NHBA samples were prepared at a protein concentration of 0.4 mg/mL in PBS buffer. Data were analyzed by subtraction of the reference data for a sample containing buffer only, using the Origin 7 software. All experiments were performed in duplicate, and mean values of the melting temperature (*T*_m_) were determined.

### Surface plasmon resonance

Surface plasmon resonance (SPR) was used to assess the binding between the huFab 5H2 and FL NHBA variants p2 (strain NZ98254), p3 (MC58), p20 (2996), and NHBA_312-427_ and point mutants thereof. All SPR experiments were performed using a Biacore T200 instrument (GE Healthcare) equilibrated at 25°C. For the single-cycle kinetics (SCK) experiments, a commercially available human Fab Capture Kit (GE Healthcare) was used to immobilize a mixture of monoclonal antibodies recognizing κ and λ subtypes of Fab fragment light chains by amine coupling on a carboxymethylated dextran sensor chip (CM5; GE Healthcare). A density level yielding ∼5000–6000 response units (RU) was achieved. The immobilized anti-human Fab monoclonal antibodies were then used to capture ∼800–1100 RU Fab 5H2 on the second of the two flow cells used. Experimental SPR running buffer contained 10 mM Hepes, 150 mM NaCl, 3mM EDTA, 0.05% (v/v) P20 surfactant, pH 7.4 (HBS-EP). *K*_D_ and kinetic parameters were calculated by performing a titration series of five consecutive injections of purified protein antigen diluted in HBS-EP at increasing concentration (range 3.125–50 nM; flow rate of 30 μL/min) followed by a single final surface regeneration step with buffer containing 10 mM glycine pH 1.7 (180 s; 10 μL/min). An anti-human Fab antibody–coated surface without captured Fab 5H2 was used as the reference channel. A blank injection of buffer only was subtracted from each curve, and reference sensorgrams were subtracted from experimental sensorgrams to yield curves representing specific binding. Data were analyzed using the standard SCK method [32] implemented by the Biacore T200 evaluation software (GE Healthcare). Each sensorgram was fitted with the 1:1 Langmuir binding model, including a term to account for potential mass transfer, to obtain the individual *k*_on_ and *k*_off_ kinetic constants. The individual values were then combined to derive the single averaged *K*_D_ values reported. SCK experiments were performed in triplicate. To assess binding of NHBA_312-427_ mutants to Fab 5H2 captured onto the sensor chip described above (capture level ~ 180 RU), single injections of purified NHBA_312-427_ mutants at a fixed concentration of 300 nM were performed with a flow rate of 10 μL/min, and surfaces were regenerated as described earlier after each cycle.

### Epitope mapping by protein microarray

A protein microarray containing recombinant overlapping fragments of NHBAp2 and NHBAp3 and nine different NHBA FL variants (p2, p3, p5, p10, p17, p20, p21, p24, p29) was generated as previously described [18, 33]. Briefly, recombinant proteins were spotted on nitrocellulose-coated slides (FAST slides, Maine Manufacturing) using the no-contact Marathon Spotter (Arrayjet, Edinburgh, UK). Non-specific binding was minimized by preincubating the protein microarray slide with a blocking solution (BlockIt, ArrayIt) for 1 hour. huFab 5H2 was diluted 1:2000 in BlockIt and overlaid on the protein array for 1 h, at room temperature. AlexaFluor®647-labelled anti-human IgG, Fab specific secondary antibody (Jackson Immunoresearch) was added for 1 h at room temperature in the dark, before proceeding with slide scanning. Fluorescence signals were detected by using a PowerScanner confocal laser scanner (Tecan Trading AG, Switzerland) and the 16-bit images were generated with PowerScanner software v1.2 at 10 μm/pixel resolution and processed using ImaGene 9.0 software (Biodiscovery Inc, CA). Elaboration and analysis of image raw fluorescence Intensity (FI) data was performed using in-house developed software and R scripts. Signals were considered as positive when their MFI value was higher than 5,000, corresponding to the MFI of protein spots after detection with AlexaFluor®647-labelled anti-human IgG, Fab specific secondary antibody (Jackson Immunoresearch) alone, plus 10 standard deviation values. The protein microarray data have been deposited to National Center for Biotechnology Information’s Gene Expression Omnibus database (https://www.ncbi.nlm.nih.gov/geo/) under series accession numbers GSE112752.

## Results and discussion

### Thermo-stability of NHBA and overall structure of fragment 133–427

In order to crystallize the vaccine antigen NHBA, we designed a construct that was predicted to lack the most disordered first N-terminal 132 amino acids, which likely impaired our previous crystallization attempts of the FL protein [34]. This construct was made of residues 133–427 (named here NHBA_133-427_) (**Fig 1A**). First, we expressed, purified and characterized this construct by differential scanning calorimetry (DSC), which showed a single melting temperature (*T*_m_) transition with a peak at 98.9°C, indicative of a very stable C-terminal β-barrel (**Fig 1B**). This remarkably high stability is also observed for the FL NHBA p20 which displays a comparable peak at 100.2°C. In addition, the FL protein shows a transition at lower temperature (54°C), suggesting the presence of a smaller N-terminal fragment containing some tertiary structure elements.

The crystal structure of NHBA_133-427_ was solved by molecular replacement (MR) at 1.8 Å resolution (**Table 1**), revealing a β-hairpin and a β-barrel encompassing residues 275–427 (**Fig 1C**). The overall architecture of the anti-parallel β-barrel (residues 312–427) is indistinguishable from that observed by NMR [15]. However, the N-terminal β-hairpin (residues N275-G305) packs against the β-barrel in a remarkably different arrangement from what was previously observed (see below) (**Fig 1D**). The crystal structure presented a dimer in the asymmetric unit (**S1A Fig**), which prompted us to study the oligomeric state of this construct in solution by SEC-MALS. This showed a monomeric state for this construct (**S1B Fig**), thus suggesting that the dimer observed in the crystal structure is merely the result of interactions that occur during crystallization.

### Novel NHBA features from structural comparisons

Structural comparison by superposition of the crystal structure of NHBA_133-427_ solved here onto the NMR structure (PDB 2LFU) [15] reveals an average root mean square deviation (rmsd) of 1.9 Å for each of the ten NMR models, on C-α coordinates, with the lowest match having an rmsd of 2.8 Å and the best one 1.3 Å. Differences were observed in the conformations of the loops of the β-barrel, suggesting local inherent flexibility. However, additional and substantial structural differences were observed in the position of the N-terminal β-hairpin, which in the crystal structure makes extensive packing interactions, mostly of hydrophobic nature, with one face of the β-barrel (**Fig 1C**). In contrast, this region in the NMR structure was previously observed to be disordered and was modeled distant from the β-barrel surface as an ensemble of 10 different flexible conformations (**Fig 1D** and **S1 Movie**). It is conceivable that this apparent flexibility of the β-hairpins arises from the use of 30 mM DPC detergent in the previous solution NMR study, which likely obstructed interactions with the β-barrel [15]. Indeed, residues involved in detergent binding were identified by chemical shift perturbation and were mapped on the surface of NHBA. This, along with observed positive ellipticities in the Phe-Tyr region of a near UV CD spectrum, previously led to the hypothesis that the exposure of Phe and Tyr was affected by detergent binding. Now, the crystal structure reveals how exposed Tyr and Phe residues in the β-barrel and the β-hairpin of NHBA are indeed exclusively located at their interface (**Fig 1E**), thus supporting the original hypothesis of detergent interference at the β-hairpin: β-barrel interface. We propose that the crystal structure represents the physiologically-relevant structure, more likely to be adopted *in situ* under detergent-free conditions.

### Anti-NHBA human Fabs and design of NHBA_312-427_

As a follow-up to the isolation of humAbs elicited by the 4CMenB vaccine and their initial characterization [18], we started structural studies with the aim to elucidate the interactions (i.e. high-resolution epitope mapping) of the N-terminal binding antibodies 12E1 and 10C3 [10] and of the C-terminal binder 5H2 (this study) to NHBA. The difficulties in generating a stable and well behaving FL or partially N-terminal truncated NHBA construct (see above the structure of NHBA_133-427_), as well as previous evidence that 5H2 bound to the C-terminal β-barrel of NHBA[18], prompted us to design a new NHBA construct containing the minimal epitope for 5H2 binding. This construct includes ß-barrel residues 312–427 only (**Fig 1A**), and in DSC shows a single peak corresponding to a melting temperature transition of ~88°C, which is ~10 degrees lower with respect to both NHBA p20 FL (100.2°C) and NHBA_133-427_ (98.9°C) (**Fig 1B**). This difference in thermo-stability might be due to the lack of the β-hairpin in construct 312–427, and thus to the solvent exposure of hydrophobic residues on the N-terminal face of the β-barrel (**Fig 1E**).

### Structures of human Fab 5H2 and of the complex Fab 5H2:NHBA_312-427_

The structure of the unbound Fab 5H2 was solved at 1.8 Å resolution by molecular replacement (**Table 1**), displaying the typical β-sandwich immunoglobulin architecture of Fabs. Three copies of Fab 5H2 were found in the asymmetric unit (**S2A Fig**). Fully refined atomic coordinates of the unbound Fab 5H2 and of the NHBA_133-427_ structure described above were used to solve (by molecular replacement) the structure of the complex Fab 5H2:NHBA_312-427_, which was refined at 2.8Å resolution. Two copies of the complex occupy the asymmetric unit, disposed orthogonally to each other (**S2B Fig**), with structural superposition indicating a very high similarity throughout all chains (rmsd of 0.4 Å for 544 aligned C^α^ atoms).

### High-resolution epitope mapping of Fab 5H2

**Fig 2:**
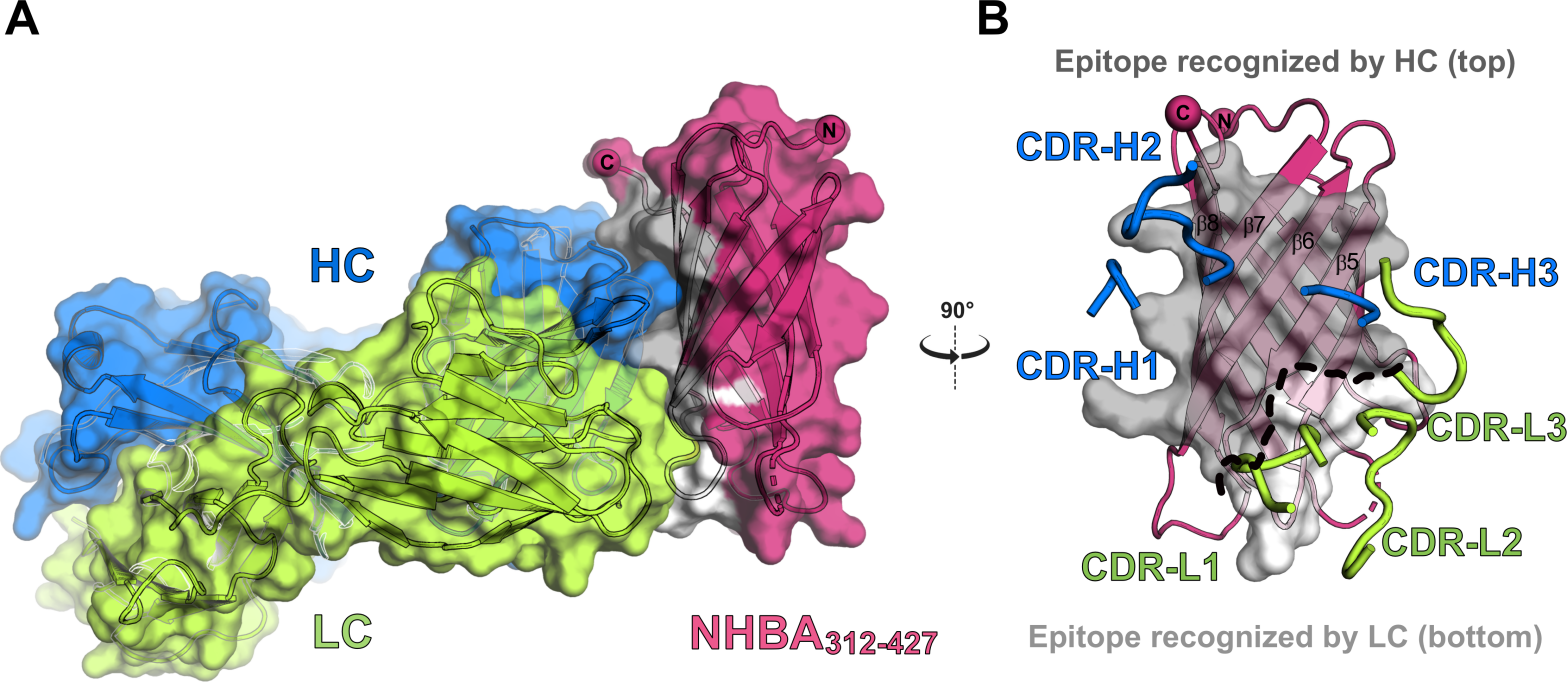
Structure of the Fab5H2:NHBA_312-427_ complex. **A**) Overall structure of the complex between Fab5H2 (H and L chain colored blue/green respectively) and the NHBA_312-427_ (colored in magenta with the epitope surface colored in grey). Buried surfaces at the epitope-paratope interface correspond to ~11% and ~4% of the total surface area of NHBA_312-427_ and Fab5H2, respectively. **B**) Front view of NHBA_312-427_ showing the five strands of its C-terminal β-barrel face that make the 5H2 epitope. The 5H2-interacting interface is colored in dark and light grey to distinguish regions contacted by 5H2 H (top) and L (bottom) chain respectively. Blue and green tubes show the 5H2 CDRs, while the rest of the Fab is omitted for clarity.

The structure of the Fab 5H2:NHBA_312-427_ complex reveals how the Fab binds to a large conformational epitope on NHBA, which is made of the entire C-terminal face of its β-barrel (**Fig 2A**). Extensive interactions are mediated by both the Fab H and L chains, with an interface that buries a total of ~1,700 Å^2^ of surface area where 5H2 complementarity determining regions (CDRs) loops fill grooves on the conformational NHBA epitope surface, creating a remarkable epitope-paratope shape complementarity. The recognition interface is dominated by contacts established by the Fab heavy chain, which contacts with all the three CDRs a surface area of ~530 Å^2^ of the antigen, while the Fab light chain interacts with NHBA by burying ~210 Å^2^ of surface area, and mostly through residues of the CDRs-L1 and L3, while L2 is almost completely excluded from the recognition (**Fig 2B**).

**Fig 3:**
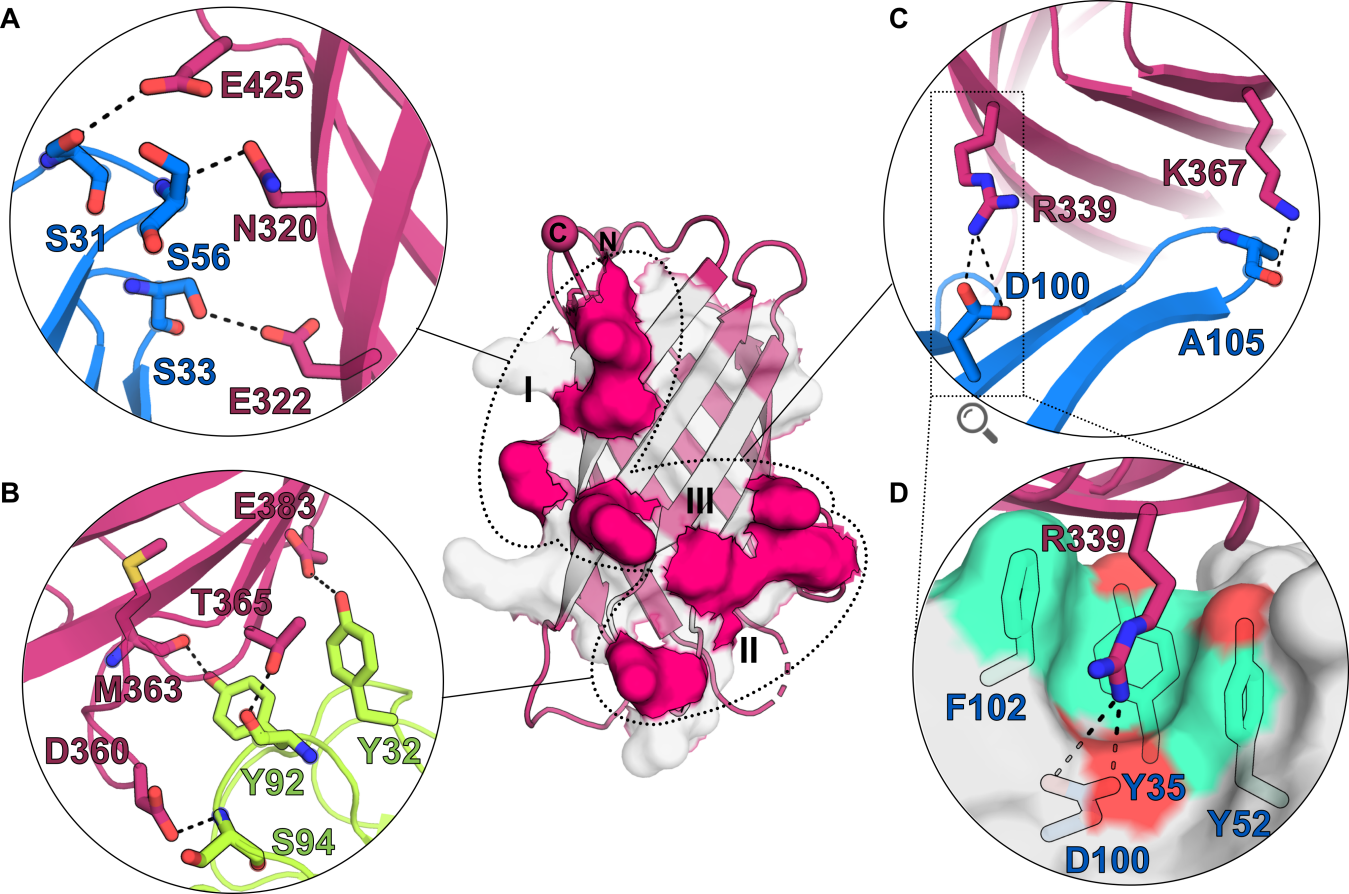
Polar contacts between Fab5H2 and NHBA. The β-barrel of NHBA, shown as dark-pink cartoon, is shown in the background in the middle of the figure. Grey surface depicts the entire 5H2 epitope, while patches of residues involved in direct bonds with Fab 5H2 are shown as solid dark-pink surfaces, and are circled and labelled. The network of polar interactions between the HC or LC of Fab 5H2 and NHBA are shown in the zoomed-in circles **A**) and **B**), respectively. **C**) Details of the patch III interactions, with zoom (**D**) into the salt bridge between NHBA R339 and Fab 5H2 D100.

Three binding patches can be distinguished on the 5H2 epitope (**Fig 3**): patch I and patch II mediate polar and hydrogen-bonding interactions with both Fab 5H2 heavy chain (patch I, **Fig 3A**) and light chain residues (patch II, **Fig 3B**); instead, patch III is centrally located and mediates two key interactions involving NHBA residues K367 and R339, with the latter forming a salt bridge with Fab 5H2 residue D100 in a peculiar arrangement (**Fig 3C**). Here, a unique paratope funnel-shaped pocket located close to the Fab HL interface traps R339 and favors its interaction with Fab 5H2 H chain D100, which seems to form a negatively charged floor surrounded by a wall of hydrophobic residues made of H chains residues Y35, Y52, and F102 (**Figs 3D** and **S3**). The distribution of the electrostatic potential on the surfaces of NHBA_312-427_ and of Fab 5H2 shows charge complementarity, with NHBA E425 facing a positively charged area contributed mainly by backbone atoms of S31 and Y55, and NHBA D356-D360 facing another positive patch on 5H2 made of side chains and backbone atoms of residues K60 and S94 (**S3 Fig**).

### Structural basis of Fab 5H2 binding to NHBA

**Fig 4:**
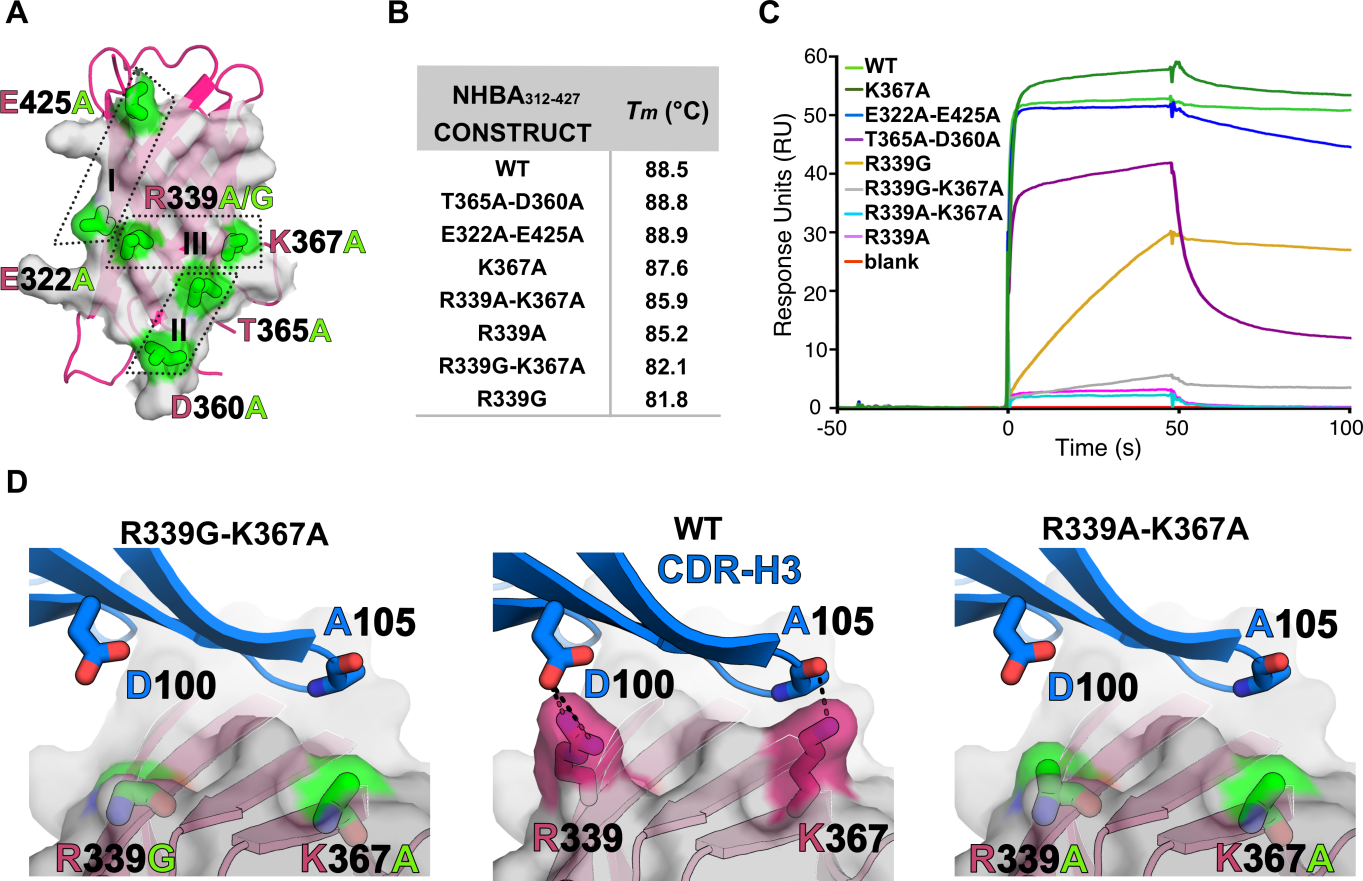
Thermostability of NHBA_312-427_ mutants and effects on 5H2 binding. **A)** Cartoon representation of NHBA_312-427_ β-barrel and surface representation of 5H2 epitope mapped in the X-ray structure, with location of the mutated sites highlighted in green and labeled. The three spots (I, II, III) mostly involved in 5H2 recognition are boxed by dotted line. **B**) *T*_m_ values of NHBA mutants as studied by DSC. **C**) Sensorgrams profiles of the SPR experiments performed using captured Fab 5H2 and NHBA_312-427_ mutants. Colors corresponding to each of the mutants are reported on the left. **D**) Zoom into region II: comparison between the direct interactions of 5H2 with NHBA_312-427_ wild type (center) and predictions of the NHBA_312-427_ mutants R339G-K367A (right) and R339A-K367A (left).

Seven NHBA mutants were generated with the goal of exploring the role of each key interaction on the 5H2 epitope. Nam ely, mutants R339A, R339G, K367A, R339A-K367A, R339G-K367A, T365A-D360A and E322A-E425A, were generated (**Fig 4A**). In order to evaluate if any of the mutants had an effect on thermo-stability, we measured their *T*_m_ by Differential Scanning Calorimetry (DSC) (**Fig 4B**). This revealed how mutations localized on the most peripheral regions of the epitope (i.e. K367A, T365A-D360A and E322A-E425A) did not significantly affect the melting temperature (*T*_m_) when compared to the wild-type NHBA_312-427_. Instead, all mutations involving residue R339 (R339A, R339G, R339A-K367A, R339G-K367A) resulted in thermal destabilizations, as inferred from decreased *T*_m_ values of up to ~7°C (**Fig 4B**). Interestingly, the highest destabilization was observed for constructs carrying the R339G mutation, either alone or in combination with K367A, and those carrying R339A, either in combination with K367A or alone. This effect can probably be attributed to the destabilizing effect that glycine has when placed in the middle of a β-strands [35–37], given also its known role in conferring conformational flexibility in proteins. These DSC analyses indicate that all constructs were folded, highly stable, and suitable for binding studies, and that the centrally-located residue R339 plays a more critical role than other residues in stabilizing the ß-barrel.

To evaluate the effects of these mutations on the 5H2 binding, SPR experiments were performed by first capturing Fab 5H2 on a sensor chip, and then injecting the seven individual NHBA_312-427_ mutants (**Fig 4C**). These experiments showed that mutations to alanine of residues E322, E425, or K367, which are involved in direct interactions with the Fab heavy chain, are not critical for 5H2 binding, while mutation of residues T365 and D360, which establish polar contacts with the 5H2 L chain, result in an altered binding profile characterized by a notably faster dissociation curve when compared to WT or other mutants. This is indicative of reduced binding, likely due to a lowered ability for the complex to remain associated after an apparently unaffected initial formation of the complex (see comparable association profiles). Other key determinants of the NHBA:5H2 interaction seem to include a combination of bonds mediated by NHBA residues R339 and K367 with the 5H2 heavy chain. Mutation of R339 to glycine results in a slower association rate, while mutation to alanine completely inhibits complex formation. The same can be observed when mutating R339 to either glycine or alanine in combination with the lysine to alanine mutation in position 367. Modeling of the R339G and R339A mutations (**Fig 4D**) helps to rationalize the effect of these mutations: the 5H2 D100 residue might contribute more repulsive interactions with alanine rather than with glycine, being alanine bulkier and marginally more hydrophobic than glycine [38], and thus resulting in residual affinity of binding.

### Fab 5H2 CDRs rearrangements upon binding to NHBA_312-427_

**Fig 5:**
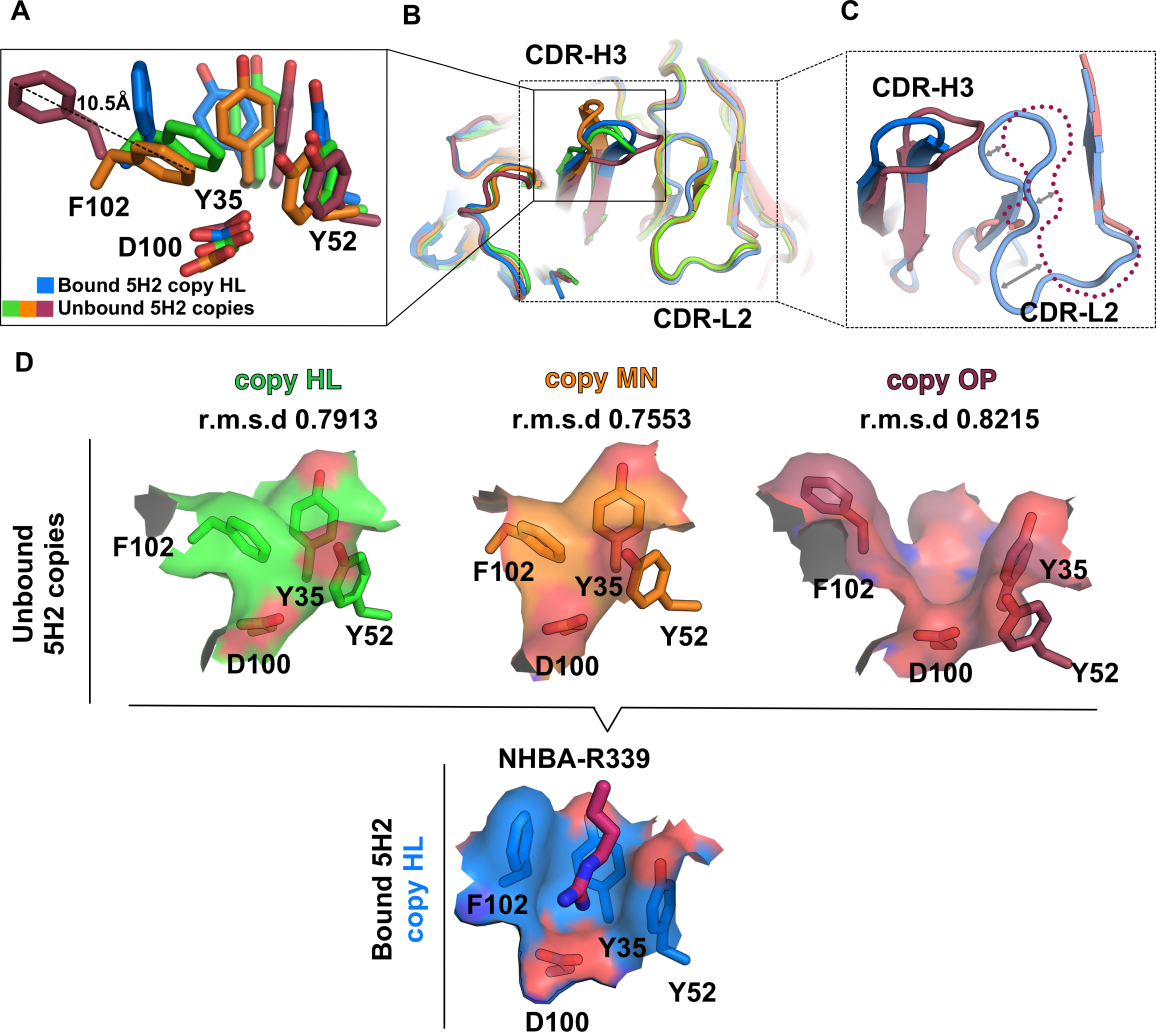
Binding-induced conformational changes in the 5H2 structures. **A**) Rotamers of residues F102, Y35 and Y52 of the unbound Fab 5H2 chains HL (green), MN (orange) and OP (raspberry), compared to those of the NHBA_312-427_-bound Fab 5H2 chains HL (blue). **B**) Top view of the superposition between unbound Fab 5H2 chains HL, MN, OP and NHBA_312-427_-bound Fab 5H2 chains HL (blue). Region with the major differences are boxed. **C**) Zoom into CDR-H3 and CDR-L2 of the unbound Fab 5H2 chains OP and NHBA_312-427_-bound Fab5H2 chains HL, where a dotted line shows the missing atoms of the CDR-L2 from the OP chains. **D**) Surfaces of the triad F102-Y52-Y35 of the unbound 5H2 structures are shown on top, compared with the NHBA_312-427_-bound 5H2 chains HL (bottom). Rmsd values of SSM between H chain variable domains of 5H2 are reported. Chain H of 5H2-bound copy was taken as reference.

The structural effects on Fab 5H2 upon binding to NHBA_312-427_ were investigated by performing structural superposition of the variable domains of the unbound and bound 5H2 structures (**Fig 5)**. This revealed local structural rearrangements upon binding that apparently result in a better fit of the interfacing antibody-antigen surface. These rearrangements are particularly evident for CDR-H3 residues Y35, Y52 and F102 of the Fab, with the side-chain of F102 displaced by ~10 Å from the unbound to the bound states (**Fig 5A**). Additional conformational changes can be observed for the 5H2 CDR-L2 loop, which likely also directly affects the nearby CDR-H3. Electron density for loop CDR-L2 (residues T48-R61) is not visible in one of the heavy chains (O) of the unbound Fab 5H2 structure, suggesting intrinsic flexibility (**Fig 5C**). Strikingly, the most evident effect of these epitope binding-mediated rearrangements is the formation of the funnel-shaped pocket that seems necessary to optimally accommodate residue R339 of NHBA, by using both shape complementarity and a mixed polar (the floor) and hydrophobic (the walls) type of interaction (**Figs 5D** and **3D**).

### Fab 5H2 elbow angles flexibility between unbound and NHBA-bound states

The availability of the unbound and bound Fab 5H2 crystal structures, and the presence of multiple copies in the asymmetric unit of both structures (**S2 Fig**), prompted us to investigate possible conformational changes of the Fab associated with NHBA_312-427_ binding. For this, we first measured the 5H2 elbow angles of the three different molecules found in the asymmetric unit of the unbound structure, observing an oscillation with values ranging from ~122° (chains HL and MN) to ~146° (chains OP) (**Table 2**). In contrast, the two asymmetric unit copies of the Fab 5H2 from the NHBA_312-427_-bound structure displayed a rather similar elbow angle of 135° and 136°, respectively. As expected, this suggests that the hinge region between C and V chains of the bound 5H2 have restricted degrees of freedom if compared to their conformations when in the unbound state, in agreement with the previously described change in elbow angle upon antigen binding [39–41]. These observations seem also in agreement with the pre-existing equilibrium hypothesis, in combination with the induced fit model as a general mechanism of antibody-antigen recognition [42]. According to the pre-existing equilibrium hypothesis, antibodies can assume a set of multiple conformations in solution that are closely related to each other, with a shift towards the antigen binding preferred conformation as soon as they engage in complex formation. This could drive the so-called early binding mechanism that is then followed by induced-fit, considered as conformational changes of the paratope upon binding [43]. For the 5H2:NHBA_312-427_ complex, we first observed a distribution of possible states from the structure of unbound 5H2, which likely reflects the intrinsic molecular motion of 5H2 even within the constraints of the crystal-packing environment. Then, it is likely that the only 5H2 conformation observed in the complex corresponds to the one better fitting the antigen surface, and thus indicating a selection of the most favorable Fab conformation.

**Table 2:** Elbow angles of Fab 5H2. Comparison between the elbow angles of the three Fab copies of the unbound 5H2 and the Fab complexed with NHBA_312-427_.

	**Unbound 5H2**			**Bound 5H2**	
**Fab chains**	HL	MN	OP	HL	IM
**Elbow angle**	122.58°	122.20 °	146.10 °	136.12 °	135.57 °

### Epitope conservation and cross-reactivity of Fab 5H2

To explain the ability of the NHBA C-terminal domain to elicit high-affinity cross-reactive antibodies, a panel of short and long epidemiologically relevant NHBA variants was chosen for sequence alignment analyses. Specifically, sequences of the β-barrels from short (p21, p17, and p10) and from long (p2, p3, p5, p24, p29) variants were aligned against the sequence of the p20 variant (also short) (**S4 Fig**). The β-barrel, and consequently also the 5H2 epitope, is made of nearly identical sequences (~98% identity across the different variants), with only slightly divergent variants being p29, p24 and p2 (sequence identities of ~96%). Interestingly, these variants contain a few point mutations exactly in the region of the 5H2 epitope; specifically, R337 is substituted with K in p24 or G in p29, and D360 is replaced by G in p2. Although D360 is directly involved in polar interactions with 5H2, the SPR data show that its substitution to G in the p2 variant does not affect the binding affinity, as also revealed by the 5H2:NHBA_312-427_ complex structure (**Table 3 and S5 Fig**). Together with the results of the binding experiments and epitope mapping, this analysis confirms the ability of Fab 5H2 to efficaciously cross-react with different NHBA variants.

**Table 3:** SPR measurements. Binfoding affinities between Fab5H2 and NHBA variants p2, p3 and p20 determined by Surface Plasmon Resonance.

NHBA Variants	k_on_ (1/Ms)	k_off_ (1/s)	U-value	K_D_ (M)
**NHBAp2 (NZ28954)**	1.0E+5	5.4E-5	5	5,2E-10
**NHBAp3 (MC58)**	4,2E+5	1.7E-4	1	4,0E-10
**NHBAp20 (2996)**	5.4E+5	2.3E-4	1	4,3E-10

Affinity constants and binding kinetics of Fab 5H2 versus different NHBA variants were calculated from measurements performed by SPR. For these, three variants of NHBA were selected, the two long variants p2 (which is the 4CMenB vaccine variant [8]) and p3, and the short variant p20. As expected, Fab 5H2 recognizes all the three NHBA variants with comparable and very high affinities, measured in the picomolar range (**Table 3**and **S5 Fig**), a feature that we can attribute to the conservation of the epitope mapped onto the C-terminal β-barrel of NHBA. Also, these binding data are in agreement with previously reported epitope mapping studies on the 5H2 cross-reactivity [18].

Finally, the ability of Fab 5H2 to cross-react with different NHBA variants was originally reported using variants p2, p3, p17, p20 and p21 studied by a combination of techniques including Protein and Peptide microarrays [18]. Here, we adopted the same Protein microarray approach, but using an enhanced panel of constructs, specifically using a total number of nine different NHBA long and short peptide variants (p2, p3, p5, p10, p17, p20, p21, p24, p29). The measured immunoreactivity of Fab 5H2 versus NHBA fragments spotted on a microarray confirmed the cross-reactive properties of Fab 5H2 also against this expanded set of variants (**S6 Fig**), again confirming the structural and sequence observations on the 5H2 epitope.

## Conclusions

Previously published structural biology studies of the 4CMenB protein antigens fHbp and NadA provided a deeper understanding of their biological functions [44–51] and introduced novel concepts for vaccine design [50]. Here, we focused on crystallographic studies of the less structurally characterized of the three 4CMenB protein antigens [8], NHBA, thus broadening the characterization of 4CMenB. In agreement with the fact that the N-terminal region of NHBA is predicted to lack secondary structures, and is thus annotated as an intrinsically disordered protein (IDP), we were unable to solve the structures of this region. It is expected that a hybrid strategy combining structural studies of the N-terminal with techniques such as small-angle X-ray scattering (SAXS) [52, 53], or by computational modeling with Rosetta [54] may cast more light on the full-length structure of NHBA. Importantly, this region also harbors important epitopes [10, 18] and may also have unknown functions mediated by disordered or motifs riven by context specific ordering. Here, in order to overcome the difficulties of crystallizing a protein containing flexible regions, we removed these regions by designing the two constructs NHBA_133-427_ and NHBA_312-427_, and we then solved their crystal structures either in the unbound/apo state or bound/complexed to the Fab derived from the humAb 5H2 that was previously isolated from subjects immunized with 4CMenB [10, 18].

The high-resolution crystal structure of NHBA_133-427_ solved here reveals a novel arrangement of a β-hairpin that in the crystal structure closely interacts with the conserved β-barrel, while in the NMR structure this was flexible and less structurally defined. In support of a putative role for the hairpin in stabilizing the barrel and thus the whole C-terminal domain, thermo-stability studies shows how NHBA_312-427_, which lacks the residues forming the β-hairpin, is destabilized by almost 10°C with respect to NHBA_133-427_. Our structure now suggests that this destabilization might be due to the solvent-exposed hydrophobic face on the β-barrel of NHBA_312-427_. We then solved the co-crystal structure of a human Fab:NHBA complex, by co-crystallizing Fab 5H2 with NHBA_312-427_. This complex structure elucidates a large conformational epitope on NHBA, which is made of an entire face of the β-barrel (the one opposite to that making interaction with the β-hairpin). This region is highly conserved across a panel of NHBA strains as confirmed by binding and protein microarray data, thus explaining its capacity to cross-react with NHBA variants from different strains. Finally, the crystal structure of the unbound huFab 5H2 also solved in this work reveals a remarkable paratope plasticity upon binding to NHBA, as well as elbow angle flexibility. Both these observations support a mechanism of pre-existing equilibrium and induced-fit for antibody-antigen (huFab 5H2:NHBA) complex formation [42].

In conclusion, we elucidated novel structural features of the vaccine antigen NHBA, the structural details of a cross-reactive epitope for a human monoclonal antibody, and the structural bases for the formation a human Fab:NHBA protein complex. These results advance our molecular understanding of the 4CMenB antigen NHBA, and at the same time contribute to the growing structural understanding of antibody-antigen complexes.

## Supporting information

S1 Movie Conformational plasticity of the NHBA N-terminal β-hairpin. One monomer from the NHBA_133-427_ crystal structure (magenta) is shown after superposition onto the NMR structure (pdb 2LFU, grey cartoon). The dashed black line connecting the two red spheres indicates the distance between residue Pro297 (depicted by the red sphere) while magenta and grey spheres show the termini of the NHBA_133-427_ crystal structure and the NMR structure (2LFU). The morph between the single state of the crystal structure and the ten states of coordinates from pdb 2LFU was performed in Pymol.
(WMV)

S1 Fig NHBA_133-427_ ASU organization and SEC-MALLS. **A)** Structure of NHBA_133-427_ showing the dimer as mixed surface/cartoon representation. **B**) SE-HPLC profile of NHBA_133-427_ construct which display a single peak at 16.6 min. **B)** SE-HPLC/MALLS profile of NHBA_133-427_. The curves plotted correspond to Absorbance Units (mAU) at 280nm wavelength (green), light scattering (red), and refractive index (blue). The numbers ‘1’ at the bottom of the gradient-shaded slice identify the beginning and end of each fraction-1, used for the MALLS analyses.
(TIFF)

S2 Fig ASU content of the unbound 5H2 and 5H2:NHBA_312-427_ structures. **A)** The overall arrangement of the three copies of 5H2 is shown, and chains are labelled. In each copy, L chains are colored with light colors (yellow, lime, salmon), heavy chains are depicted with dark colors (orange, green, raspberry). **B)** The two copies of the Fab5H2:NHBA_312-427_ complex are depicted with the 5H2 chains HL colored blue/green and chains IM colored violet/pale green. NHBA molecules binding to HL and IM are colored magenta and raspberry, respectively. On the bottom of each structure a schematic representation of the ASU copies relative orientation.
(TIFF)

S3 Fig Electrostatic potential of the complex interface. Open book view of the interfacing Fab5H2 paratope **A**) and NHBA epitope **B**) surfaces. Circles with the same layout represent complementary regions. Surfaces are colored according to the electrostatic potential distribution, which was calculated with APBS {Lerner M. G., 2006 #406} where red and blue surfaces show negative and positive charges as contoured in the range from –3 kBTe-1 (red) to +3 kBTe-1 (blue), while white surfaces show neutral potentials.
(TIFF)

S4 Fig Multiple sequence alignment of the C-terminal b-barrel of NHBA variants. A panel of NHBA short (p21, p17, p10) and long (p2, p3, p5, p24, p29) variants from *N*. *meningitidis* strains NM117, GB013, M12923, NZ98254, MC58, M18017, M01820 and M16686 were aligned against NHBA p20 (strain 2996). Black background indicates fully conserved residues, grey background indicates not 100% conservation. The 5H2 epitope is highlighted in yellow, while green triangles show the non-conserved residues of the 5H2 epitope. Above the alignment, the arrows represent the position of each β-strand, according to the X-ray structure solved in this work. The numbering scheme refers to NHBA p20 variant.
(TIFF)

S5 Fig SPR sensorgrams of Fab5H2 and NHBA p2, p3, and p20 variants. Surface plasmon resonance (SPR) was used to determine the dissociation constants (K_D_), using the single cycle kinetic (SCK) approach, for the NHBA variants p2, p3 and p20. The titrations included NHBA concentrations from 3.125–50 nM. Colored curves represent the experimental data, black lines represent the fitted curves.
(TIFF)

S6 Fig Immunoreactivity of Fab5H2 to a panel of recombinant NHBA fragments spotted on a microarray. Each horizontal bar represents a protein or protein fragment in the microarray aligned along the NHBA sequence and color-coded from light grey to dark red according to mean fluorescence intensity (MFI) values, as shown in the vertical bar. The protein microarray data are available under accession number GSE112752 at the National Center for Biotechnology Information’s Gene Expression Omnibus database.
(TIFF)
