# Supplementary figures and images for: Structures of NHBA elucidate a broadly conserved epitope identified by a vaccine induced antibody
Source: PLoS One. 2018 Aug 22;13(8):e0201922. doi: 10.1371/journal.pone.0201922 (PMC6104945; doi:10.1371/journal.pone.0201922)

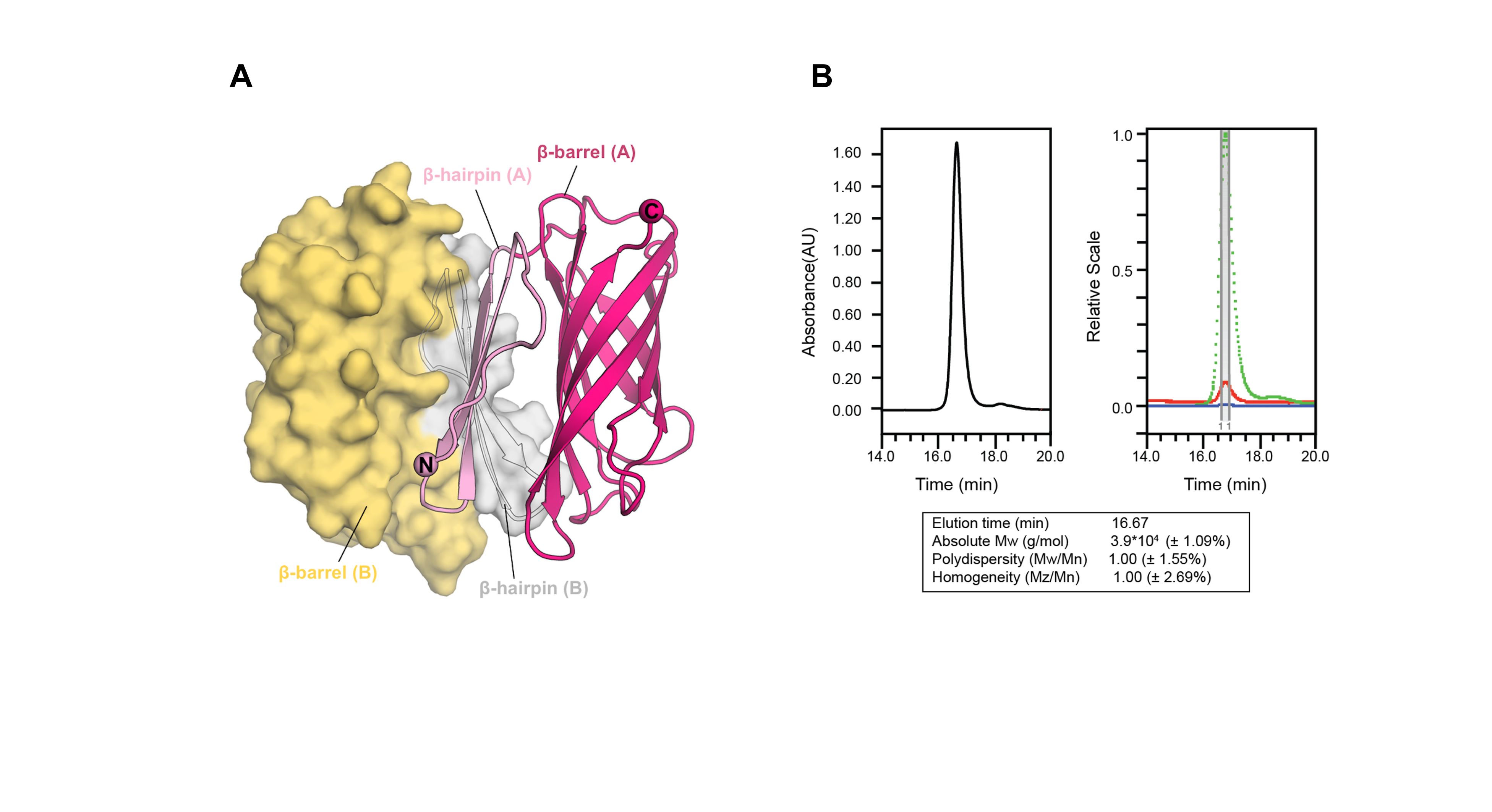

Supplement: S1 Fig — A) Structure of NHBA133-427 showing the dimer as mixed surface/cartoon representation. B) SE-HPLC profile of NHBA133-427 construct which display a single peak at 16.6 min. B) SE-HPLC/MALLS profile of NHBA133-427. The curves plotted correspond to Absorbance Units (mAU) at 280nm wavelength (green), light scattering (red), and refractive index (blue). The numbers ‘1’ at the bottom of the gradient-shaded slice identify the beginning and end of each fraction-1, used for the MALLS analyses. (TIFF) [file pone.0201922.s002.tiff]

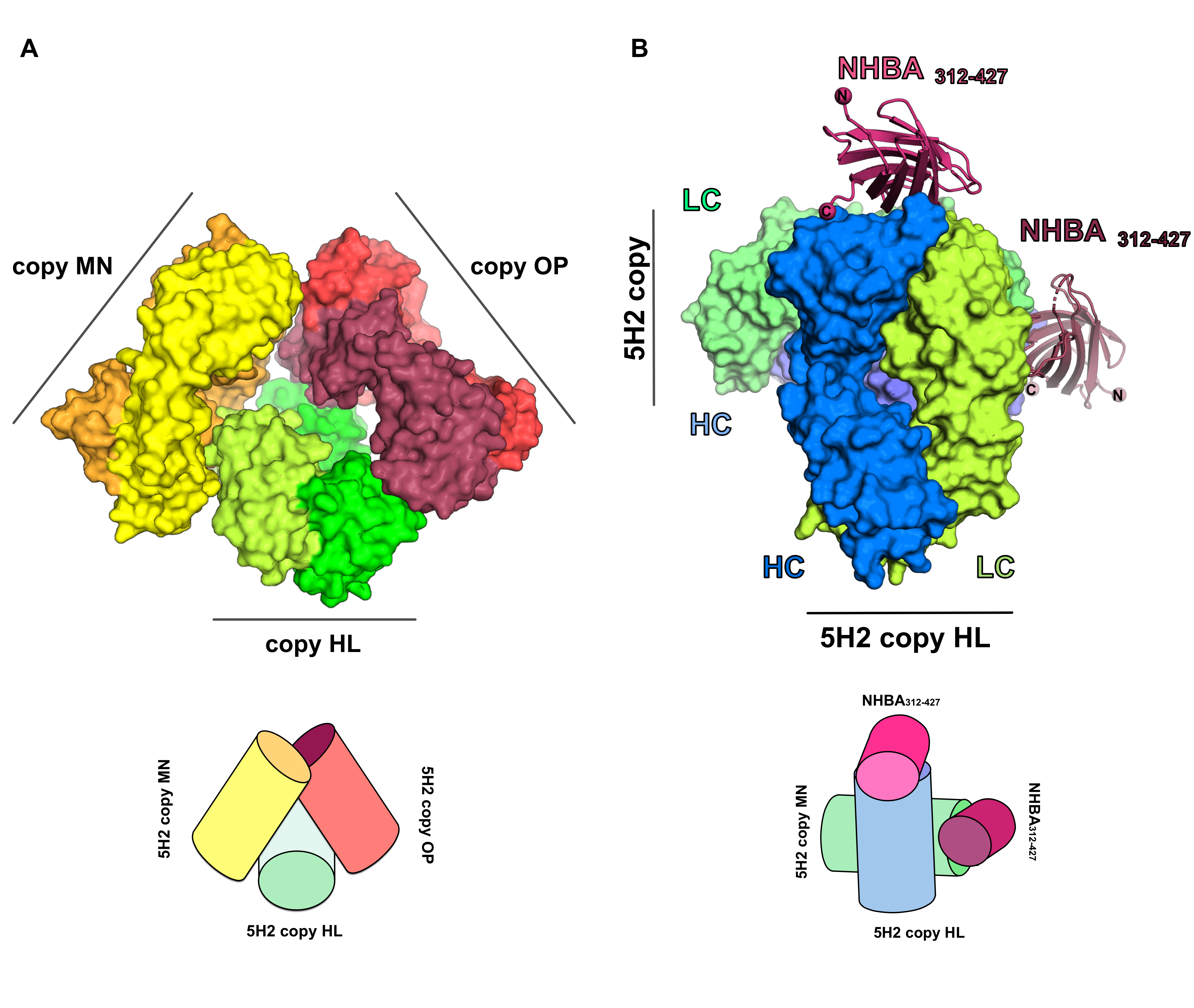

Supplement: S2 Fig — A) The overall arrangement of the three copies of 5H2 is shown, and chains are labelled. In each copy, L chains are colored with light colors (yellow, lime, salmon), heavy chains are depicted with dark colors (orange, green, raspberry). B) The two copies of the Fab5H2:NHBA312-427 complex are depicted with the 5H2 chains HL colored blue/green and chains IM colored violet/pale green. NHBA molecules binding to HL and IM are colored magenta and raspberry, respectively. On the bottom of each structure a schematic representation of the ASU copies relative orientation. (TIFF) [file pone.0201922.s003.tiff]

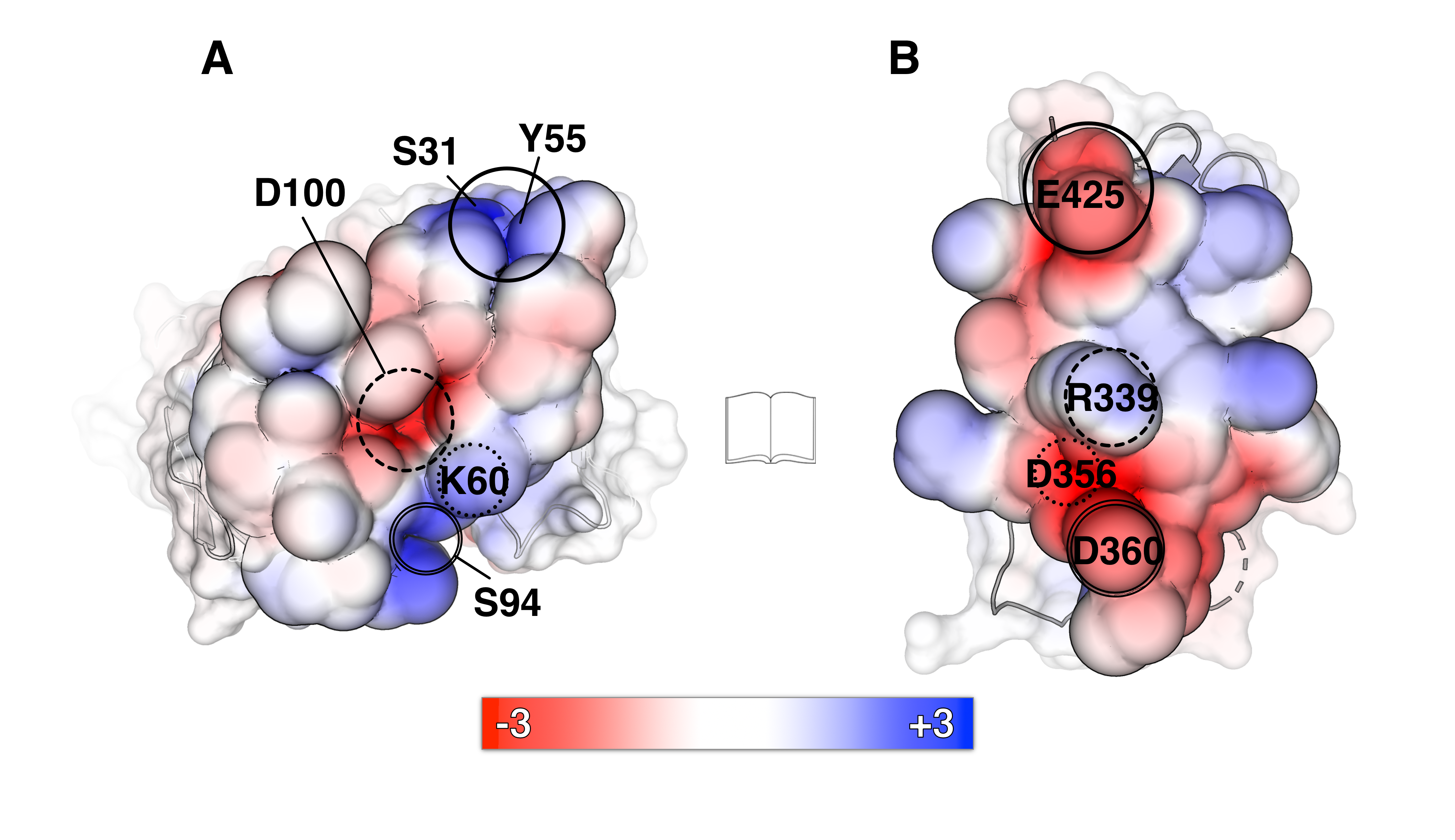

Supplement: S3 Fig — Open book view of the interfacing Fab5H2 paratope A) and NHBA epitope B) surfaces. Circles with the same layout represent complementary regions. Surfaces are colored according to the electrostatic potential distribution, which was calculated with APBS {Lerner M. G., 2006 #406} where red and blue surfaces show negative and positive charges as contoured in the range from –3 kBTe-1 (red) to +3 kBTe-1 (blue), while white surfaces show neutral potentials. (TIFF) [file pone.0201922.s004.tiff]

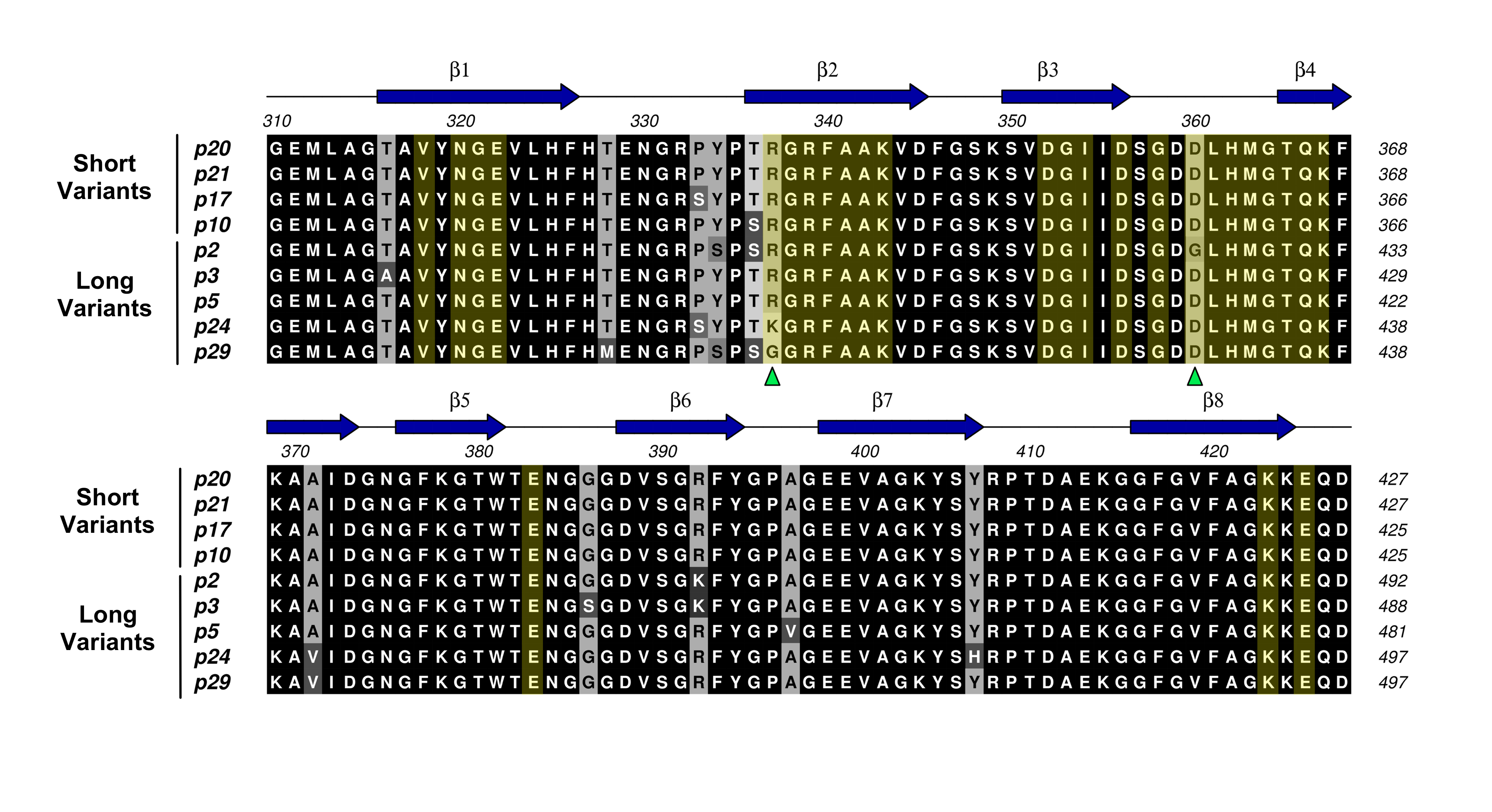

Supplement: S4 Fig — A panel of NHBA short (p21, p17, p10) and long (p2, p3, p5, p24, p29) variants from N. meningitidis strains NM117, GB013, M12923, NZ98254, MC58, M18017, M01820 and M16686 were aligned against NHBA p20 (strain 2996). Black background indicates fully conserved residues, grey background indicates not 100% conservation. The 5H2 epitope is highlighted in yellow, while green triangles show the non-conserved residues of the 5H2 epitope. Above the alignment, the arrows represent the position of each β-strand, according to the X-ray structure solved in this work. The numbering scheme refers to NHBA p20 variant. (TIFF) [file pone.0201922.s005.tiff]

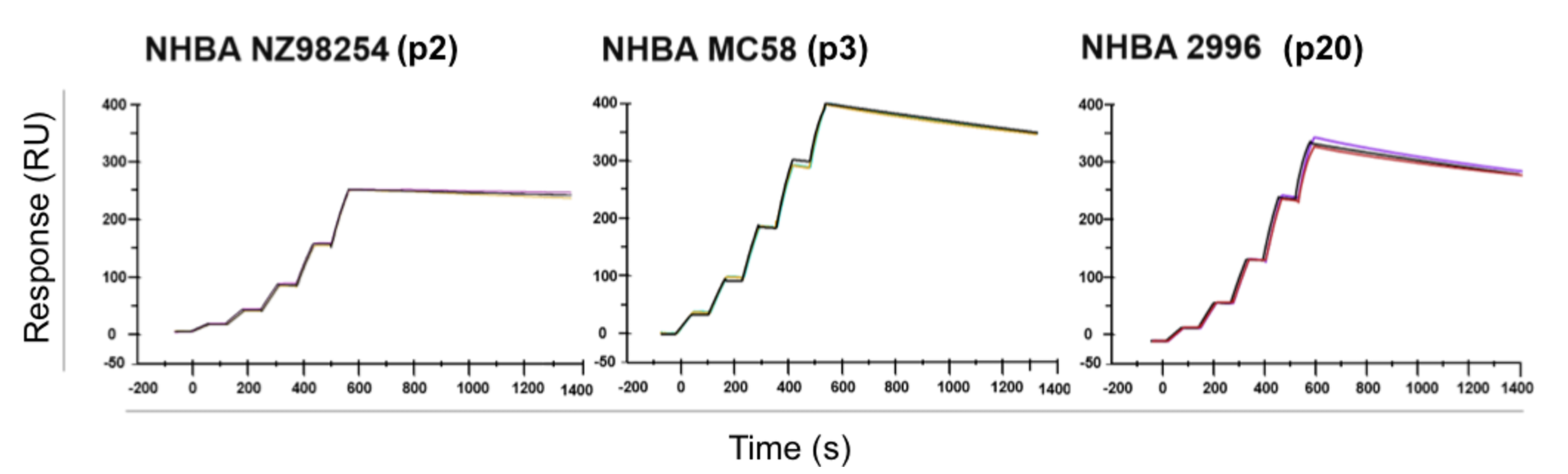

Supplement: S5 Fig — Surface plasmon resonance (SPR) was used to determine the dissociation constants (KD), using the single cycle kinetic (SCK) approach, for the NHBA variants p2, p3 and p20. The titrations included NHBA concentrations from 3.125–50 nM. Colored curves represent the experimental data, black lines represent the fitted curves. (TIFF) [file pone.0201922.s006.tiff]

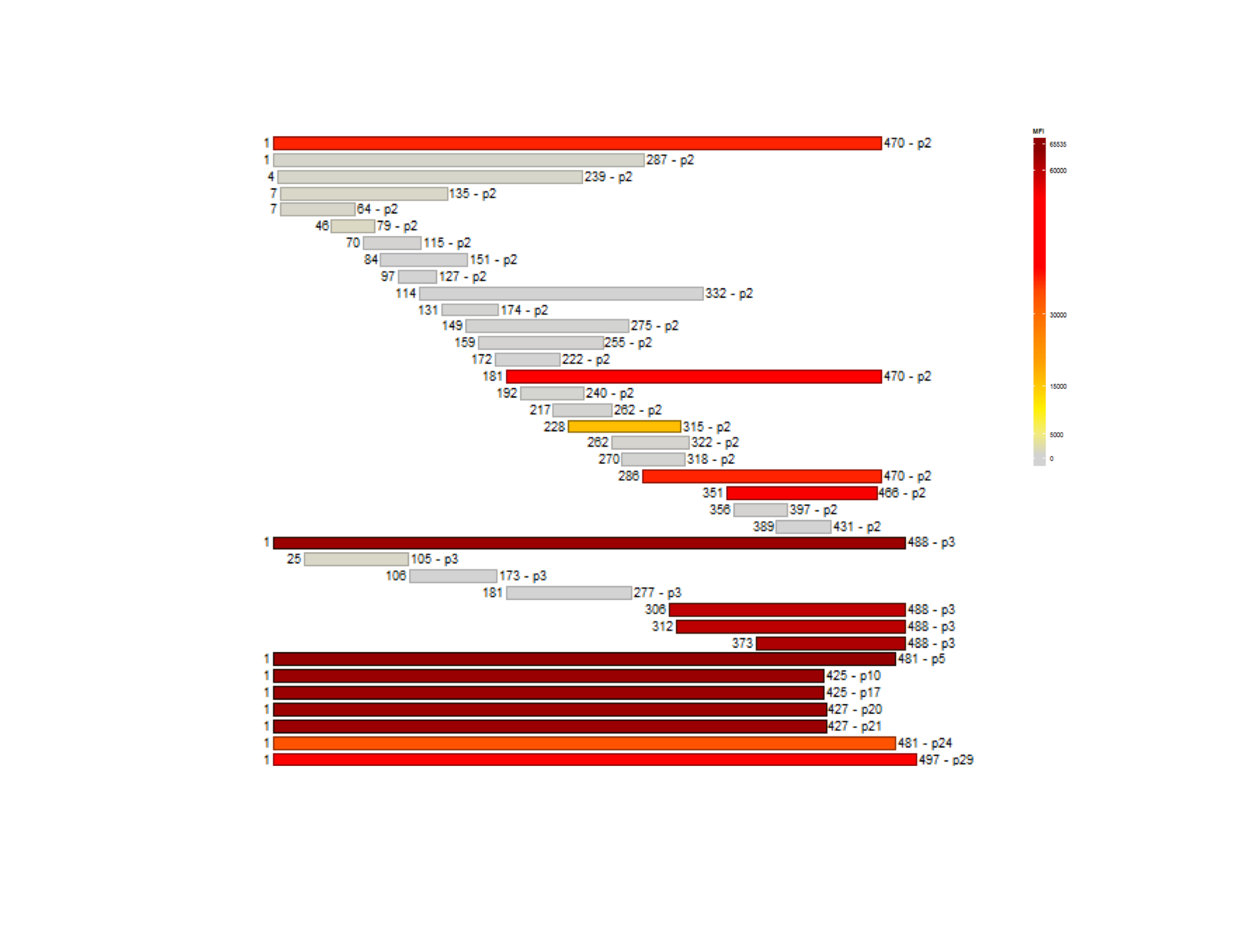

Supplement: S6 Fig — Each horizontal bar represents a protein or protein fragment in the microarray aligned along the NHBA sequence and color-coded from light grey to dark red according to mean fluorescence intensity (MFI) values, as shown in the vertical bar. The protein microarray data are available under accession number GSE112752 at the National Center for Biotechnology Information’s Gene Expression Omnibus database. (TIFF) [file pone.0201922.s007.tiff]
